# Was millennial scale climate change during the Last Glacial triggered by explosive volcanism?

**DOI:** 10.1038/srep17442

**Published:** 2015-11-30

**Authors:** James U.L. Baldini, Richard J. Brown, Jim N. McElwaine

**Affiliations:** 1 Department of Earth Sciences, University of Durham, Durham, DH1 3LE, UK

## Abstract

The mechanisms responsible for millennial scale climate change within glacial time intervals are equivocal. Here we show that all eight known radiometrically-dated Tambora-sized or larger NH eruptions over the interval 30 to 80 ka BP are associated with abrupt Greenland cooling (>95% confidence). Additionally, previous research reported a strong statistical correlation between the timing of Southern Hemisphere volcanism and Dansgaard-Oeschger (DO) events (>99% confidence), but did not identify a causative mechanism. Volcanic aerosol-induced asymmetrical hemispheric cooling over the last few hundred years restructured atmospheric circulation in a similar fashion as that associated with Last Glacial millennial-scale shifts (albeit on a smaller scale). We hypothesise that following both recent and Last Glacial NH eruptions, volcanogenic sulphate injections into the stratosphere cooled the NH preferentially, inducing a hemispheric temperature asymmetry that shifted atmospheric circulation cells southward. This resulted in Greenland cooling, Antarctic warming, and a southward shifted ITCZ. However, during the Last Glacial, the initial eruption-induced climate response was prolonged by NH glacier and sea ice expansion, increased NH albedo, AMOC weakening, more NH cooling, and a consequent positive feedback. Conversely, preferential SH cooling following large SH eruptions shifted atmospheric circulation to the north, resulting in the characteristic features of DO events.

Millennial scale climate change is one of the most characteristic and yet enigmatic features of recent glacial intervals. From 30 to 80 ka BP, glacial baseline conditions were interrupted by 21 abrupt climate change events, termed Dansgaard-Oeschger (DO) events, associated with warm interstadial conditions in Greenland but colder conditions in Antarctica. The apparent temperature shifts were locally dramatic; for example, DO Event 8 (DO-8) occurred 38.17 ka BP, and was characterised by 11.8 °C Greenland warming^1^, which is comparable to warming across a full glacial-interglacial transition. Additionally, evidence from high resolution Greenland ice cores suggests that Greenland warming can occur extremely rapidly, potentially within 1 to 3 years^2^. Any explanation for DO events must therefore account for both the rapidity and magnitude of the observed temperature change. Similarly, any cause must also explain the apparent hemispheric asymmetry characteristic of DO events; Greenland warming was associated with Antarctic cooling^3^, a phenomenon known as the ‘bipolar see-saw’^3^. The hemispheric asymmetry is not restricted to high latitudes. Increasing speleothem-based evidence indicates that DO events were associated with substantial atmospheric reorganisation at low latitudes, with a strengthening of the East Asian Summer Monsoon (EASM) system^4^,^5^ in the Northern Hemisphere (NH) and a weakening of the South American Monsoon (SAM) and the Australasian Monsoon (AM) in the Southern Hemisphere (SH)^6^,^7^. Explanations for the asymmetric hemispheric response to DO events often invoke Atlantic Meridional Overturning Circulation (AMOC) shifts possibly induced by meltwater pulses^7^,^8^,^9^, but this interpretation is not universally accepted, and other proposed explanations include solar forcing^10^, internal ocean-atmosphere oscillations^11^, and volcanism^12^. Because the substantial climate shifts associated with DO event initiation can seemingly occur on human timescales, identifying the underlying cause of such abrupt climate change and considering these forcings in a modern context is critical.

Here we use published ice core (synchronised on the AICC2012 chronology^13^), volcanological, and speleothem-based evidence to argue that DO events were triggered by asymmetrical hemispheric cooling caused by explosive SH volcanic eruptions. Conversely, we argue that very large NH eruptions forced abrupt Greenland cooling events and, under the right conditions, were associated with large ice rafting events (Heinrich Stadials). This perspective is consistent with recent results suggesting that ice rafting events were a consequence of, but did not trigger, NH cooling^14^. Large explosive volcanic eruptions are conventionally thought to result in global cooling for several years following the eruption, based on the concept that stratospheric volcanogenic sulphate aerosols reflect solar radiation and cool the planet essentially uniformly. Large low latitude eruptions are therefore often considered the most climatologically significant because low latitudes receive a disproportionately high amount of insolation^15^, although in-depth studies highlight considerable complexities in the climate response to eruptions based on ejecta volume, sulphate content, explosivity, and latitude^16^,^17^ (see Supplementary Information). However, global cooling does not seem to have occurred following several large, well-documented Pleistocene eruptions. For example, the Toba supereruption at ~74 ka BP is followed by lower NGRIP δ^18^O (Greenland cooling) but higher EDML δ^18^O values (Antarctic warming), thereby providing no support for homogeneous global cooling^18^. Similarly, evidence from Lake Malawi in east Africa does not show any evidence for substantial cooling at low latitudes^19^. The apparent absence of ‘volcanic winter’ in response to the largest eruption of the Quaternary is enigmatic when considered from the conventional perspective. Similarly puzzling is the apparent strengthening of the SAM and AM and concomitant weakening of the EASM coinciding with the Toba eruption (Fig. 1).

However, very recent research focussing on the last 500 years has demonstrated the importance of asymmetric hemispheric cooling induced by sulphate aerosol injections^20^,^21^ on atmospheric circulation. Observational, modelling, and proxy studies now firmly implicate 20^th^ Century anthropogenic sulphate aerosol emissions with greater NH cooling compared to the SH, resulting in southward migration of the Intertropical Convergence Zone (ITCZ), drying at NH low latitudes, and more rainfall at SH low latitudes^22^,^23^. Recent research also demonstrates that this same asymmetric cooling effect occurred following the injection of sulphate aerosols into the stratosphere following large volcanic eruptions over the last 100 years (based on instrumental data)^20^ and 500 years (based on stalagmite rainfall proxy data)^21^. These studies collectively demonstrate that over the last few centuries NH aerosols (volcanogenic and anthropogenic) forced the ITCZ to the south by cooling the NH relative to the SH, and that SH eruptions forced northward ITCZ migration. The atmospheric and temperature response associated with the Toba eruption is therefore consistent with the recently detected climate response to more recent (but far smaller) NH eruptions. We suggest that the Toba eruption initiated southward ITCZ migration by inducing a NH-SH temperature asymmetry, which resulted in more SAM rainfall but reduced rainfall in the NH low latitudes (e.g., the EASM), consistent with speleothem-based evidence (Fig. 1), and identical to the response to NH eruptions over the last 500 years. A southward displaced ITCZ would have shifted Hadley circulation cells to the south, compressing the SH Polar Cell, forcing the SH Polar Front southward, and resulting in locally warmer Antarctic temperatures (Supplementary Information). The opposite response would have occurred in the NH, where atmospheric reorganisation would have resulted in extreme and sudden cooling in Greenland and extension of NH glacial and sea ice, characteristics associated with the abrupt transition between DO-20 and Greenland Stadial (GS) 20 occurring at that time. We propose that the positive feedbacks following NH eruptions (e.g., NH sea ice expansion, NH continental glacier expansion, increased NH albedo, and AMOC weakening) prolonged the climatic response to the Toba eruption by several hundred years, not dissimilar to recent results suggesting that Little Ice Age cooling was initiated by NH volcanism in the 13^th^ Century but was sustained over hundreds of years by a positive feedback involving sea ice and oceanic circulation^24^. The timing of the Toba supereruption is consistent with the abrupt cooling into GS20, although this seems superimposed on a cooling trend that began ~100 years earlier, potentially linked to decreasing insolation. However, the Toba eruption is nearly indistinguishable from the inception of Antarctic warming (Figs 1 and 2) (Supplementary Information). We suggest that large NH eruptions that occurred during interstadials abruptly ended Greenland interstadial conditions and promoted a rapid transition to stadial conditions, which were favoured during much of the Last Glacial because of low atmospheric greenhouse gas concentrations and insolation conditions. Conversely, large NH eruptions that occurred during already cold conditions resulted in less Greenland cooling but led to glacier extension and Heinrich Events. This is supported by the established association of the 39.280 ± 0.110 ka BP Campanian Ignimbrite supereruption of the Campi Flegrei supervolcano with GS9 and Heinrich Event 4 (HE4)^25^. We also note that the inception of HE5a at ~53 ka BP is indistinguishable from the timing of the magnitude (M) 7 Ischia eruption (53 ± 3.3 ka BP). Additionally, all eight very large (M ≥ 7; Tambora-sized or larger) Pleistocene NH eruptions (see Supplementary Information for selection criteria used) are within error of a NH cooling event (Fig. 3), and Monte Carlo simulations demonstrate that this relationship is significant at the 95% confidence level (Supplementary Information) (Fig. 4).

The stratospheric sulphate loading associated with large SH eruptions could have had the opposite effect, preferentially cooling the SH relative to the NH, shifting the ITCZ to the north, compressing the NH Polar Cell, and shifting the NH Polar Front to the north (Supplementary Information). We suggest that this northward shift in the polar fronts in response to large SH eruptions resulted in dramatic warming across the North Atlantic and NH glaciated regions, promoting sea ice collapse and the partial retreat of continental ice sheets, all characteristic features of DO events. The presence of large continental ice sheets would have provided a strong positive feedback. The proposed northward NH Polar Front migration would have caused continental ice sheet retreat, consequently reducing albedo, destabilising NH permafrost, and resulting in subtle methane increases characteristic of DO events^26^. This is consistent with inferred minor perturbations to the carbon cycle during DO events^26^, with the suggestion that atmosphere methane that accumulated during DO events had a high latitude source^27^, and with ice core evidence suggesting a substantial northward shift in methane source region following DO event initiation^28^. The flux of radiocarbon-depleted carbon into the atmosphere during DO events, often interpreted as AMOC strengthening and increased outgassing of radiocarbon depleted-bottom waters, may also originate from permafrost thaw, as previously suggested for the Bølling/Allerød^29^. The lower albedo and strengthened AMOC following NH ice sheet decay following SH eruptions could have produced a positive feedback that prevented the short-term re-establishment of continental ice sheets to their full glacial extent as favoured by still low atmospheric CO_2_ concentrations. This caused a gradual return to pre-eruption conditions hundreds or even thousands of years after the initial sulphate forcing, unless the slow process was expedited by a large NH eruption (forcing an abrupt return to stadial conditions, as was the case following the Toba eruption) or the cooling process was interrupted by another SH eruption, as may have occurred after an unknown SH eruption triggered DO-13 at 49.5 ka BP (Fig. 2).

Very recent research using ice core data from a high snow accumulation rate site in Antarctica suggests that that the temperature signal associated with DO events originated in the NH and was then transmitted to the SH ~200 years later by oceanic processes^30^. Interestingly, the authors of the study highlight the fact that the North Atlantic processes thought to have initiated DO events could themselves have resulted from a remote and ‘elusive’ trigger. We propose that this trigger was a SH eruption, which initially cooled the SH for just 1–5 years due to increased atmospheric aerosol loading. Existing proxy records are too low resolution to detect this initial aerosol-induced cooling, but the cooling was sufficient to shift atmospheric circulation to the north, warming NH high latitudes, promoting NH ice sheet decay, strengthening AMOC, and initiating a positive feedback mechanism. This NH warming could then have been propagated back to the SH ~200 years later, well after the initial volcanic aerosol-induced cooling effects had abated. NH warming (triggered by a SH eruption) would have resulted in ice shelf collapse, increased freshwater outputs to the North Atlantic, or reduced sea ice extent, all previously suggested mechanisms driving DO events^30^,^31^. These other processes could have resulted from and amplified the initial volcanic forcing, possibly by inducing AMOC shifts which were propagated to the south ~200 years later^30^.

We focus on the interval from 30 to 80 ka BP because of the high density of millennial-scale climate oscillations within that interval, and because the proposed mechanism would be most effective during intervals of time characterised by intermediate ice volume and 65°N insolation, such as most of this interval (see Supplementary Information) (Fig. 5). Unfortunately, the volcanological catalogue of known eruptions prior to 10 ka BP is very incomplete because: (*i*) erosion removes volcanic deposits from the geologic record and increases uncertainty associated with the estimation of eruption magnitude, (*ii*) burial by younger deposits hides older deposits from study, and (*iii*) many volcanoes still remain understudied or even unknown. Even over only the last one thousand years, instances exist where evidence of large eruptions is apparent in ice core volcanogenic sulphate records but the volcano responsible has only recently been identified (e.g., Rinjani, 1257 A.D.) or is still unknown (e.g., the 1809 A.D. eruption). Prior to the Holocene, large eruptions from SH calderas are particularly underrepresented (see Supplementary Information).

Despite the apparent undercount and chronological uncertainties, over the interval from 30 to 80 ka BP all five radiometrically-dated large (M ≥ 6; Pinatubo-sized or larger) SH volcanic eruptions are within chronological uncertainty of the initiation of a discrete DO event. Too few radiometrically-dated SH eruptions exist to confidently assess the link with DO events, but previous research linked direct evidence of SH eruptions derived from optical dust logger data from Antarctic Siple Dome ice with DO events over the period 27 to 70 ka^12^. The link between SH volcanism and DO events was significant at the 99% confidence level, although a well-defined mechanistic link was absent. We propose that this ‘missing link’ is the now apparent hemispheric temperature asymmetry induced by stratospheric volcanic aerosols, followed by an ice/albedo and ocean circulation feedback. Future research should test the hypothesis further by improve the dating of both climate and volcanological records, and by using isotope-enabled GCM modelling.

This hypothesis not only clarifies the mechanisms that forced abrupt millennial scale climate change during glacial conditions, but also helps predict future change. Large, explosive eruptions may indeed lead to low latitude drought in the hemisphere of the eruption, but our model suggests additional consequences that merit further consideration. For example, an extremely large NH eruption might cause nearly global cooling, but may result in localised SH high latitude warming by forcing the SH Polar Front to the south. A sufficiently large atmospheric disruption could initiate or accelerate the destabilisation of the West Antarctic Ice Sheet. Paradoxically, very large eruptions could therefore not only cause substantial mean global cooling, but also high latitude warming (in the hemisphere opposite the eruption), ice sheet collapse, and catastrophic sea level rise.

## Additional Information

**How to cite this article**: Baldini, J. U.L. *et al.* Was millennial scale climate change during the Last Glacial triggered by explosive volcanism? *Sci. Rep.* **5**, 17442; doi: 10.1038/srep17442 (2015).

## Supplementary Material

Supplementary Information

## Figures and Tables

**Figure 1:**
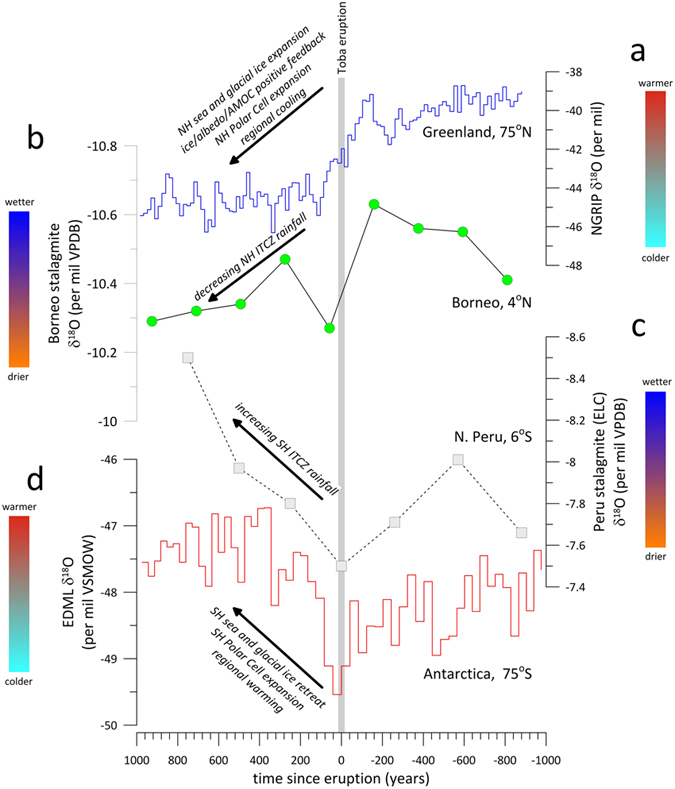
Low latitude atmospheric circulation and high latitude temperature records spanning the time interval during which the 74 ka Toba super-eruption occurred. (**a**) The NGRIP ice core δ^18^O record, (**b**) the SC03 stalagmite δ^18^O record from Secret Cave, Gunung Mulu National Park, Borneo^32^, (**c**) the El Condor Cave (ELC) stalagmite δ^18^O record from northern Peru^6^, and (**d**) the EDML δ^18^O record from Antarctica^33^. The records are arranged by latitude. The grey box indicates the timing of the Toba supereruption at 73.72 ka BP. The ice core records are synchronised on the AICC2012 timescale, and both stalagmite records are dated independently using ^230^Th dating.

**Figure 2:**
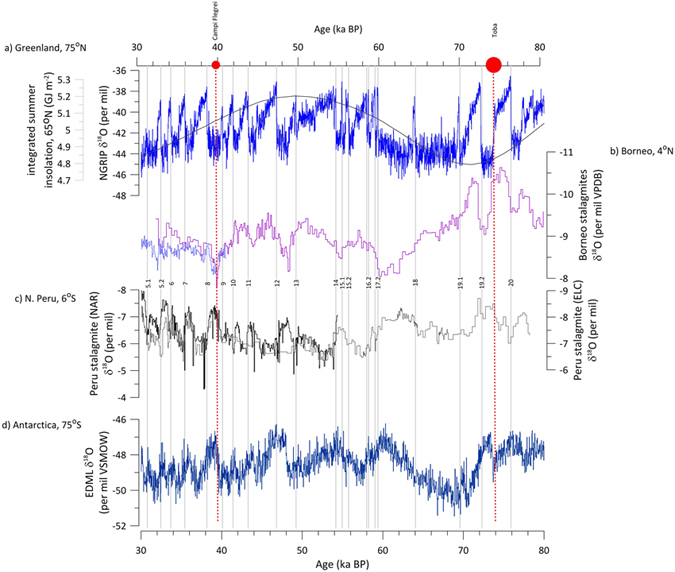
Low latitude atmospheric circulation and high latitude temperature records over the interval 30 to 80 ka BP. (**a**) The NGRIP ice core δ^18^O record^34^ and integrated summer insolation for 65°N^35^ with a τ (melting threshold) = 275 W m^−2^, (**b**) the SC03 stalagmite δ^18^O record from Secret Cave, Gunung Mulu National Park, Borneo^32^, (**c**) δ^18^O records from the El Condor Cave (ELC) and the Cueva del Diamante (NAR) stalagmites from northern Peru^6^, and (**d**) the EDML δ^18^O record from Antarctica^33^. The numbered grey boxes highlight the timing of DO events. The red circles indicate the timing of the two highest precision NH volcanic eruptions over this time interval, the Campanian (Campi Flegrei)^25^ and Toba^18^ eruptions. No comparably well-dated SH volcanic eruptions exist over this time interval. Please see Supplementary Information for the timing of all radiometrically-dated eruptions over this time interval. Error bars on the NH eruption dates (2σ) are smaller than the symbols used.

**Figure 3:**
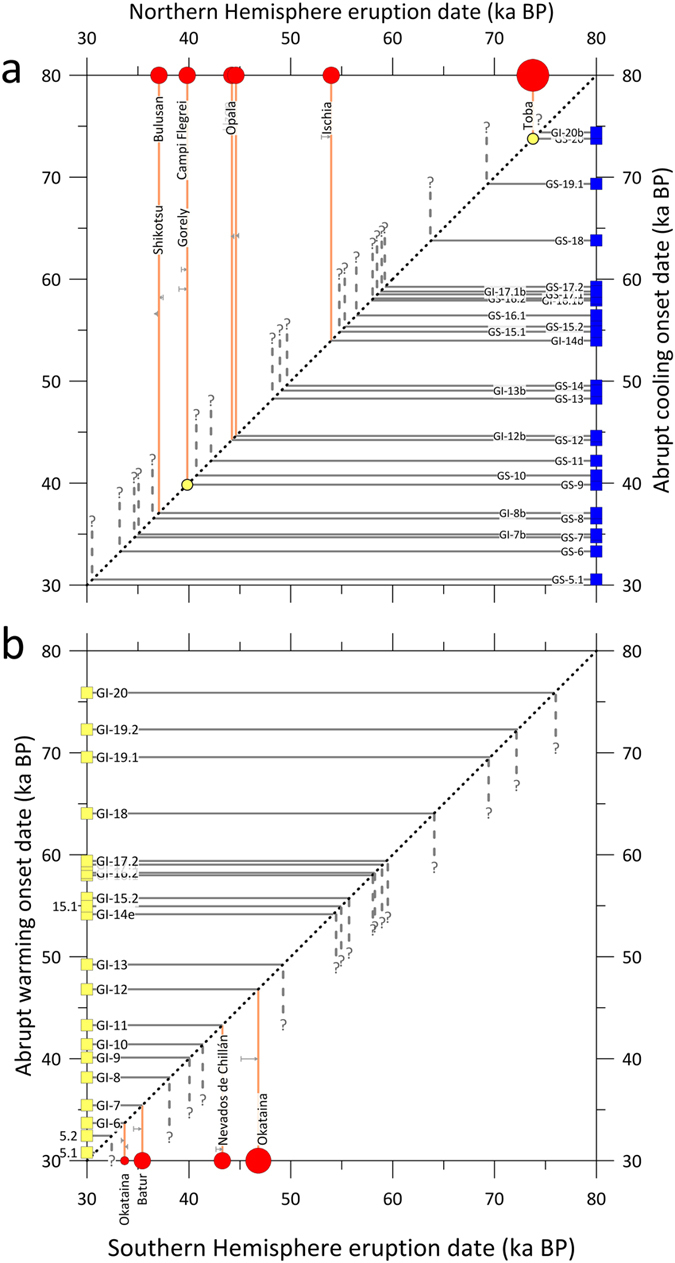
Possible correlations between millennial scale climate shifts and known volcanic eruptions. (**a**) All known very large (M ≥ 7) NH volcanic eruptions with radiometric ages (red circles) shifted within error to previously documented rapid cooling events (blue squares). (**b**) Known large radiometrically-dated (M ≥ 6) SH volcanic eruptions (red circles) shifted within error to previously documented Greenland warming events (yellow squares). The small grey horizontal arrows in both panels represent the total shift required to obtain a match to an abrupt climate event. All eight NH eruptions occur within error of an abrupt Greenland cooling event, and all five SH eruptions timings are within error of a warming event. Dashed lines with question marks represent abrupt climate shifts possibly linked to yet unidentified eruptions. The label ‘Okataina’ at 33.5 ka BP refers to two distinct eruptions responsible for Okataina Units K and L. The two yellow circles indicate previously published correlations.

**Figure 4:**
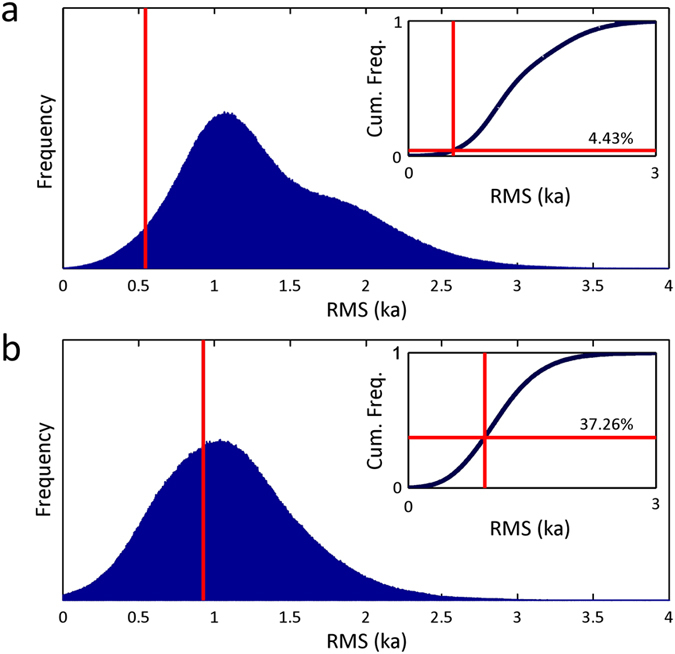
Results of Monte Carlo simulations assessing the significance of the link between (a) NH and (b) SH eruption age distribution and abrupt climate change over the interval 30–80 ka BP. The root-mean-square (RMS) statistic is the summation of the distances between a set of eruption ages and the nearest abrupt climate change events (abrupt Greenland cooling events for NH eruptions, and warming events for SH eruptions) (see Supplementary Information). A set of eight NH eruption dates was randomly selected from a uniform distribution ten million times, and a set of five SH eruptions dates selected ten million times, and the RMS calculated. The blue frequency distributions illustrate the results of these simulations, and the vertical red lines illustrate the RMS of the actual eruption ages. The insets show the cumulative frequency distributions.

**Figure 5:**
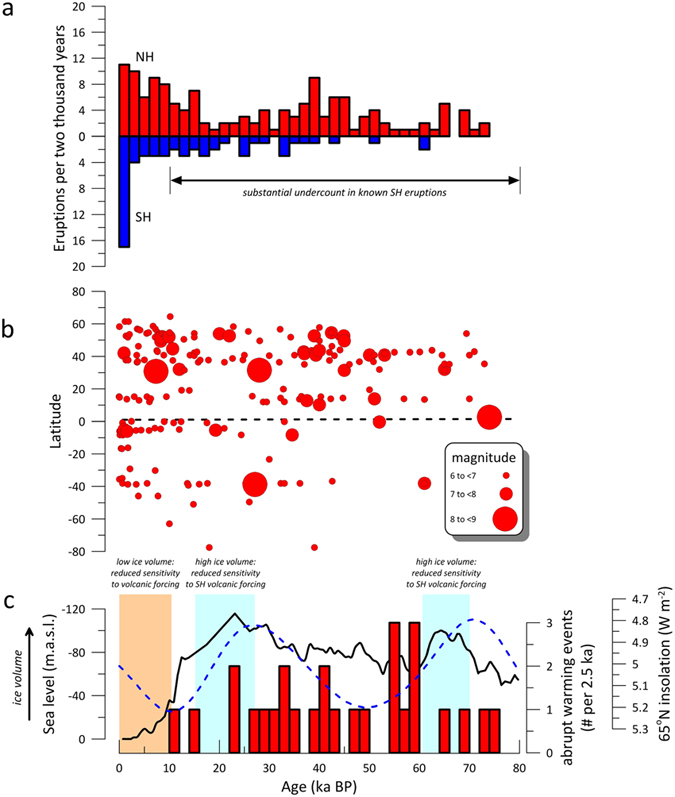
Global volcanic eruption history over the last 80 ka, based on data in the LaMEVE database, and ice volume conditions. (**a**) Number of Magnitude ≥6 (Pinatubo-sized or larger) NH and SH eruptions per two millennia from present to 80 ka BP. The low number of known SH volcanic eruptions prior to the last two ka reflects a significant undercount in known eruptions. (**b**) Latitude and ages of known Magnitude ≥6 volcanic eruptions from present to 80 ka BP^36^. (**c**) A histogram of abrupt Greenland warming events^37^, the Red Sea sea level reconstruction (interpreted as reflecting global ice volume)^38^ (solid black line), and 65°N insolation^35^ (dashed blue line). Intervals characterised by high ice volume and low insolation hypothesised as relatively insensitive to SH eruptions are highlighted in blue; intervals with very low ice volume lacking the positive feedback required to sufficiently amplify volcanic eruptions are highlighted in orange.
