# Supplementary material for: Was millennial scale climate change during the Last Glacial triggered by explosive volcanism?
Source: Sci Rep. 2015 Nov 30;5:17442. doi: 10.1038/srep17442 (PMC4663491; doi:10.1038/srep17442)
Supplement: Supplementary Information [file srep17442-s1.pdf]

Supplementary Information for "Was millennial scale climate change during the Last Glacial triggered by explosive volcanism?"

James U.L. Baldini[1], Richard J. Brown[1], and Jim N. McElwaine[1]

[1]Department of Earth Sciences, University of Durham, Durham, DH1 3LE, UK.

Supplementary Methods

Timing of millennial scale shifts

In this research we refer to both the Greenland and Antarctica ice core data on the AICC2012 chronology[1] (with ages reported as thousands of years before 1950) to facilitate the direct comparison between the records. This chronology is the same as the GICC05 chronology for the Greenland records until 60 ka BP, but minor differences exist over the interval older than 60 ka BP. Greenland Interstadial (GI) and Greenland Stadial (GS) event start timing is available in numerous publications, although subtle discrepancies exist between different estimates. Here, we use the very comprehensive estimates by Rasmussen et al. (2014)[2] resulting from the INTIMATE project efforts, reported on the GICC05 chronology. These dates are based on the synchronisation of several Greenland ice cores, and formally define abrupt climate change within the Last Glacial. The Greenland Interstadial (GI) initiation dates are referred to as Dansgaard-Oeschger Event initiation dates, and are interchangeable[2]. However, here we also discuss Antarctic ice cores, and slight differences between the GICC05 and AICC2012 chronologies exist across the older portion of the interval discussed. We therefore derive our own estimates for the start dates for GS-20, GS-21.1, and DO-20 based on the NGRIP $\delta^{18}O$ data on the AICC2012 chronology, which synchronises Greenland

and Antarctic ice core records based on methane concentrations and the presence of shared volcanic sulphate peaks. This produces a very minor difference between these three dates and the Rasmussen et al. (2014) dates; for example, for DO-20 we use a date of 75.8864 ka BP compared to the Rasmussen et al. date of 76.390 ka BP, a difference of ~0.5 ka. We note that this difference only applies to the date of three events (the oldest three) and that for the 41 other events discussed the dates used are those of Rasmussen et al. (2014). Errors associated with ice core chronologies at 60 ka BP are approximately 1.7 ka (Svensson et al., 2012 and 2013).

The timing of the ~74 ka Toba super eruption

The timing of the ~74 ka BP Toba super eruption is controversial despite it being the largest volcanic eruptions of the Quaternary. The timing of the Toba eruption relative to the ice core proxy records (e.g., the date does not take into account errors in the ice core chronology) used here is from Svensson et al (2013) and is based ice core sulphate peaks, annual layer counting, and atmospheric gas variations found in Antarctic and Greenland ice cores[3]. Svensson et al. identified several candidate sulphate peaks appearing in ice cores from Greenland and Antarctica that occurred at approximately the time of the Toba eruption, based on direct radiometric ages of the Youngest Toba Tuff (YTT). Of these peaks, the authors suggested that a peak occurring at 2547.97 metres depth in the NGRIP core (their 'T2') is the most likely candidate sulphate peak associated with the Toba eruption. We therefore use the depths of this peak in the NGRIP and EDML cores as the timing of the Toba eruption with respect to the ice cores, which is 73.75 ka BP according to the AICC2012 chronology.

The absolute eruption age, used relative to the stalagmite records from Borneo and Peru, is based on the $^{40}Ar/^{39}Ar$ dating of the YTT produced by Mark et al. (2014)[4] of 75.0±0.9 ka BP,

which is the most recent high-precision date available. However, this date is slightly older than another recently published YTT $^{40}Ar/^{39}Ar$ date of 73.88 ± 0.32 ka BP by Storey et al. (2012)[5]. Mark et al. suggest that this discrepancy is partly due to differences in astronomical tuning and calibration of $^{40}Ar/^{39}Ar$ between the two dates. They suggest that if the same (recently updated) calibration techniques they used are applied to the Storey et al (2012) age estimation, it would change to 74.2 ± 0.4 ka BP, which is within error of their 75.0 ka BP date. For the stalagmite records, an eruption of the magnitude of Toba should have left some impression on the stalagmite records from Borneo. We therefore agree with a previous suggestion[6] that the presence of a large isotopic excursion in the Borneo record is the result of the proximal eruption. Consequently, we suggest that the Toba eruption occurred at 74.37 ka BP relative to the stalagmite records, a date essentially indistinguishable from the Storey et al (2012) date (revised by Mark et al (2014)). The date we use is only 170 years older than the revised Storey et al (2012) date, and its use rather than the revised Storey et al (2012) date makes no difference to the interpretations discussed here. The slightly younger date used in the ice core records here for the eruption (73.75 ka BP, based on sulphate spikes in the ice core[3]) relative to the $^{40}Ar/^{39}Ar$-based age estimate used in the stalagmite ages (74.37 ka BP) suggests that the ice core chronology may be slightly too young at ~74 ka BP, an interpretation supported by recently published correlations between NGRIP and stalagmite data[7].

Selection of eruptions to include in analysis

The success of the analysis relies on complete objectivity in the selection of the climate events as well as the volcanic eruptions. The eruptions were not selected arbitrarily, and the priority was choosing eruptions where both the magnitude estimate and dating accuracy was

high. In other words, our analysis would not be correct if we had used a large number of eruptions with dubious age estimates. With this in mind, the criteria we used for selecting the eruptions were straightforward: i) The eruption appears in the Large Magnitude Explosive Volcanic Eruptions (LaMEVE) database. LaMEVE is the most comprehensive and most up-to-date explosive eruption dataset available. It contains information on over 1,800 Quaternary eruption records including eruption ages and erupted volumes. Data from the scientific literature have been checked for quality, and then compiled into the database. This eliminates subjectivity on our part, because the eruptions included are checked independently for quality, although we have also gone back to the original literature to double-check accuracy. Where appropriate, we have used radiometric data from very recent literature that has not yet been incorporated into LaMEVE. ii) For NH eruptions, we chose to use only Magnitude 7 eruptions because these are the best-dated, but also because errors exist associated with the estimation of magnitude of all eruptions, and these are more important for smaller eruptions. For example, if we had included Magnitude 6 eruptions, not only would the age uncertainties been greater, but their magnitude estimate might have been inaccurate as well, and the eruption might actually have been a Magnitude 5 with few climatic repercussions. On the other hand, the magnitude of Magnitude 7 eruptions is better constrained (they are larger eruptions and have attracted more research) and even if the estimate were inaccurate for any given eruption, it is very unlikely that the real magnitude would be less than Magnitude 6 (it is an exponential scale), which would probably still have climatic effects. iii) For Southern Hemisphere eruptions, we chose eruptions that were Magnitude 6 or above for the simple reason that there were far fewer eruptions in the SH than in the NH, and if we had used a threshold of M7 we would have eliminated all eruptions. iv) For both NH and SH eruptions, the eruptions needed to have a radiometric date. This is critical, because we do not wish to include eruptions that are not well-dated. The selection of the largest, most well-dated

eruptions from an independent database seems to us to be the best way to objectively seleft eruptions for our analysis.

Supplementary Discussion

Volcanological controls on climate

The climatological effects of volcanic eruptions are diverse and still only partially understood. The principal effect of explosive volcanic eruptions on climate is summer cooling of the troposphere. When exposed to solar radiation, sulphur dioxide released by volcanic eruptions is oxidised to form sulphate. In the stratosphere, these sulphate aerosols scatter solar radiation and cool the troposphere and lower latitudes, although the aerosols appear to warm the stratosphere itself. The veil of volcanic sulphate aerosols reflects incoming solar shortwave radiation[8], but tends to only reside in the atmosphere for approximately 1-3 years[9]. Low latitude eruptions have traditionally been perceived as exerting a stronger radiative cooling effect on climate[9] because aerosols produced by those eruptions may spread to both hemispheres with greater ease[10] and because sulphate aerosols may reflect more incoming solar radiation in low latitude regions that receive higher annually averaged insolation. However, high latitude eruptions may also affect climate through a combination of radiative and dynamical effects[11, 12]. High- and mid-latitude eruptions alter global atmospheric circulation patterns by preferentially cooling the hemisphere of the eruption, inducing hemispheric temperature asymmetry that results in reorganisation of atmospheric circulation[13, 14]. Specifically, the ITCZ may migrate away from the hemisphere of the eruption, influencing the position of atmospheric circulation cells in both hemispheres and ultimately potentially affecting the latitude of the polar fronts. Research using ozone measurements over recent decades has demonstrated that the polar fronts shift in response to

Hadley Cell position, and that polar front repositioning occurred following recent volcanic eruptions[15], although this effect is still poorly researched. A strong link between the ITCZ and polar fronts also seems to exist over the Holocene[16, 17] and since the Last Glacial Maximum[18], and suggests that polar front latitude in each hemisphere is sensitive to the latitude of the downward limb of the Hadley Cell in that hemisphere. Although abundant empirical data now supports this inter-hemispheric teleconnection, the precise physical mechanism leading to such an atmospheric reorganisation following volcanic eruptions is still unclear. It may involve wholesale meridional shifts in atmospheric circulation cells prompted by inter-hemispheric temperature differentials, or may involve a more complex mechanism, possibly via a series of teleconnections involving North Atlantic temperature's effects on the ITCZ, the South Pacific Split Jet, and the South Pacific subtropical front, as described in Chiang et al. (2014)[19]. Furthermore, eruptions may trigger climate change by affecting Atlantic Meridional Overturning Circulation (AMOC). Numerous previous publications have proposed that shifts in AMOC strength are the fundamental forcing behind abrupt climate change during the Last Glacial (e.g. Alley et al. 2007[20]), and it is possible that the volcanic trigger proposed here acts to strengthen (in the case of SH eruptions) or weaken (in the case of NH eruptions) AMOC, which then continues to affect climate over longer timescales. Atmospheric reorganisation following volcanic eruptions might directly affect AMOC, or may first trigger glacier and sea ice shifts that subsequently affect AMOC.

The magnitude of an eruption (i.e., the volume of ejecta injected into the atmosphere) is clearly linked to the potential of that eruption to affect climate[11]. However, erupted volume is not the only variable controlling the climatic significance of an eruption, and a variety of other factors are important, such as sulphate content, explosivity, time of year, and latitude[9, 11, 21, 22]. For example, sulphur contents in magma vary over several orders of magnitude[23], and bulk sulphur content of an eruption is a fundamental variable controlling the strength of the

radiative effects caused by that eruption. Recent volcanological evidence suggests that eruption magnitude is not always proportional with its sulphur content; for example, the millennium eruption of Changbaishan (A.D. ~941) was one of the largest eruptions of the last 2,000 years by magnitude but appears to only have had minor climatological effects due to its low sulphur content[24, 25]. Evidence for past volcanogenic sulphur is preserved within ice cores, although issues with sampling resolution (potentially missing the eruption entirely) and time-averaging (reducing the apparent size of an eruption) may complicate detection of eruptions using ice cores and often does not provide definitive information regarding sulphur contents of individual eruptions[24]. Additionally, smaller eruptions of very S-rich magma could produce similar climate effects as larger volume eruptions of less S-rich magma[23]. Estimates of sulphur content of individual eruptions based on volcanic rocks are uncertain, because much of the sulphur exists as volatiles, which are degassed and lost to the atmosphere. Bulk SO_2 emissions from an eruption is therefore an additional uncertainty that is not necessarily (but often is) linked to the magnitude of the eruption. Similarly, definitively linking a sulphate spike preserved in glacial ice to a large, globally-significant eruption rather than a smaller, more proximal one is difficult, particularly in Greenland ice cores that are often affected by Icelandic volcanism. These issues greatly complicate assigning individual sulphate spikes in Greenland ice cores to individual climate events. This issue is well-illustrated by the large number of candidate peaks potentially associated with the relatively-well researched ~74 ka BP Toba eruption[3]; ice core evidence for smaller eruptions is even more ambiguous.

The time of year of the Toba super eruption may have affected the climatic response. Although Toba is a low latitude NH volcano close to the equator, the hemispheric distribution of aerosols will depend partially on the position of the ITCZ and therefore on the time of year. If the Toba eruption occurred during NH summer, the ITCZ may have been located to

the north of Toba, and SH Hadley circulation could have entrained aerosols arising from the eruption and transported them predominantly to the south. Conversely, if the eruption occurred during NH winter, the ITCZ would have been located to the south of Toba, and aerosols transported preferentially to the north by NH Hadley circulation. This is consistent with available satellite evidence of aerosol distributions following modern eruptions, as well as with the observed response of the ITCZ following historical eruptions (Ridley et al., 2015). More extensive NH ice sheets at the time of the eruption would have shifted mean ITCZ position further to the south than its modern mean position, so the probability of the ITCZ having been to the south of Toba at the time of the eruption is high. It is important to note however that a low latitude position promotes a more even hemispheric distribution of sulphate aerosols compared with high latitude eruptions regardless of ITCZ position, and future research should assess the importance of latitude on hemispheric temperature asymmetry.

Sensitivity to intermediate ice volume conditions

Asymmetrical hemispheric cooling due to volcanological sulphate aerosol injections into the stratosphere is increasingly viewed as a powerful mechanism controlling atmospheric circulation in both hemispheres, resulting in dramatic climate change at critical atmospheric boundaries, such as in areas affected by the ITCZ and the polar fronts. We suggest that the proposed response to either NH or SH eruptions is most pronounced during periods characterised by intermediate ice volume conditions. Whereas large NH eruptions could have forced substantial NH ice sheet extension during the Last Glacial (partly through the proposed ice/albedo/AMOC feedback), the lack of substantial ice sheets on North America or Europe prevented this from occurring during the Holocene or other low ice volume intervals.

Conversely, during the coldest intervals of the last 100 ka (e.g., from ~20-30 ka BP and ~60-70 ka BP) a combination of substantial ice volume, reduced insolation, and low CO_2 may have mitigated the climatic effects of SH eruptions, explaining the lack of substantial DO events. Only after integrated summer insolation (65°N)[26] began to increase were SH eruptions again able to force DO events.

We therefore restrict our analysis to the interval 30-80 ka BP, which is characterised by intermediate ice volume and 65°N insolation conditions (other than from ~60-70 ka BP) and abundant millennial-scale climate oscillations. This interval of time is also directly comparable with that chosen by Bay et al. (2004) (25-70 ka BP). We have chosen to extend our analysis back to include the Toba eruption, which is the largest (and best dated) eruption of the Quaternary. We have not analysed the period 10-20 ka BP because complexities associated with deglaciation (e.g., changing insolation and atmospheric pCO_2 concentrations) may obfuscate the volcanic forcing. The interval from 20-30 ka BP is not considered because of high ice volume and low insolation.

Volcanological dating and preservation

Information on the magnitude, age, and location of eruption used in this study is derived primarily from the Large Magnitude Explosive Volcanic Eruptions (LaMEVE) database (http://www.bgs.ac.uk/vogripa/index.cfm) and references therein, which contains records for >1880 Quaternary eruptions of Magnitude 4 and above harvested from other peer-reviewed scientific literature and other databases. About one third of those eruptions were dated using either historical, radiometric or proxy dating techniques[27]. Dating methods vary from low precision (stratigraphic) to much higher precision radiometric methods (e.g., radiocarbon, Ar-Ar). More tightly constrained proxy ages are derived from tephra layers or sulphur layers

contained within lacustrine varves or ice cores, although it is often difficult to finger-print individual sulphur layers in ice cores. The most commonly used radiometric techniques are K-Ar and ^{40}Ar/^{39}Ar, particularly for eruptions >50 ka old. These techniques are based upon the decay of ^{40}K to ^{40}Ar and measure time since the radiogenic Ar became trapped in the rock (typically measured in crystals or groundmass), which is taken as the most recent cooling event. They rely on a number of assumptions (see Kelley, 2002[28]). Ar-Ar is more precise than K-Ar, but precision and accuracy are dependent on the uncertainty of the age of mineral standards[28]. Errors implicit with the Ar-Ar technique mean that derived ages for tephra layers erupted ~30-70 ka ago typically have error bars of several hundred to several thousand years, depending on the quality and quantity of the material measured. However, although the precision quoted is often quite high, the accuracy depends on sampling and analysing sufficient amounts of material as well as calibration techniques. An excellent example of issues concerning the accuracy of some radiometric ages concerns the dating of the Rotoiti eruption of the Okataina volcano (New Zealand). The eruption was dated as having occurred at 61.071 ± 1.4 ka BP based on K/Ar and ^{40}Ar/^{39}Ar ages of material at a distal site[29]. However, this date has since been refined using combined ^{238}U/^{230}Th disequilibrium and (U–Th)/He zircon dating approach, and a new date of 45.1 ± 3.3 ka BP produced. Here we use the latter age determination, but the ~16 ka differences between dates with precisions of ± 3.3 ka or better illustrates the issues involved in even radiometric dating of Quaternary eruptions.

The preservation of volcanic ash layers in the geological record decreases with increasing geological time. Erosion, burial, and regrowth of vegetation are the key processes that remove ash layers from the visible geological record[27] and reduce their apparent volume. The net effect is an apparent decrease in frequency of eruptions back through geological time[27] (Fig. 5, main text), coupled with a reduction in the apparent volume. Under-recording is more marked for smaller eruptions than for larger eruptions: ~70% of M7 or larger eruptions have

been recorded[27]. However, large eruptions at coastal or island volcanoes have a poor preservation potential due to dispersal of products over the sea, and tropical climates promote strong erosion and vegetation regrowth. Highly productive volcanoes can quickly bury earlier deposits under thick sequences of younger ash layers.

Finally, the completeness of the catalogue of past eruptions varies substantially geographically. The catalogue is more complete for Northern Hemisphere eruptions due to the bulk of the Earth's landmass being concentrated north of the equator. Additionally, availability of research resources influences the completeness of the volcanological record, with Europe, North America, and Japan having the most complete records[27]. Remote, inaccessible or predominantly coastal/island volcanoes, such as those in Indonesia, are much more poorly catalogued.

High erosion rates and dense vegetation characteristic of equatorial regions contribute to the issue of poor preservation of low latitude coastal/island volcanoes. We suggest that this under-representation may also reflect flooding of Glacial-aged Indonesian calderas by the ~125 m deglacial sea level rise, leaving these completely submerged. The Indonesian archipelago currently consists of over 18,000 islands with over 120 active volcanoes, and it is reasonable that more volcanic islands existed during the LGM sea level low stand. This concept is supported by recent satellite-derived bathymetry evidence identifying large underwater volcanoes within the region as well as with abundant evidence for a significant undercount in Pleistocene eruptions; assuming the volcanological records are complete over the last century and eruption frequency is constant through time, only 0.005% of all Indonesian eruptions $M \geq 2$ are known for the period 5-20 ka[30]. The record of larger volume eruptions appears more complete, but based on analysis of the LaMEVE database by us and by others[27], over 600 large ($M \geq 6$ (1991 Pinatubo eruption-sized)) SH eruptions and over 30 extremely large ($M \geq 7$ (1815 Tambora eruption-sized)) SH eruptions are missing from the

volcanological record over the period 0-60 ka. Additionally, errors implicit in radiometric methods currently used to date Pleistocene eruptions, and conflicting published eruption ages, make correlating specific eruptions to specific climate events problematic. Our hypothesis is therefore testable, but requires excellent dates for both abrupt climate transitions and volcanic eruptions; existing errors are substantial, and represent the most substantial obstacle to definitively ascribing abrupt climate change during the Last Glacial to large volcanic eruptions.

Monte Carlo simulations and assessing significance

The probability that volcanic eruption date distribution is associated with abrupt climate change as manifested in the NGRIP record (AICC2012 chronology) was evaluated by considering the null hypothesis that uniformly distributed eruption dates are independent of the climate change events. Two slightly different models were tested: *i)* using a fixed number of eruptions corresponding to the number of well-dated eruptions across the interval of interest and *ii)* with a variable number of eruptions, chosen from a Poisson distribution with the same mean number of eruptions as *i)*. No substantive differences between these two approaches were found.

To evaluate the probability of non-randomness, we define the root-mean-squared (RMS) best match statistic:

$$s = \sqrt{\frac{1}{N^e}\sum_{k=1}^{N^e} \min_j \left(T_j^c - T_k^e\right)^2}$$

where N^e is the number of eruptions, T_k^e are the eruption dates, and T_j^c are the dates of abrupt climate change events (e.g., DO events or abrupt stadial onsets). The probability distribution function and the cumulative distribution function of this statistic were generated by using 10 million random samples (for each hemisphere) (Fig. 4, main text). In other words, 10 million simulations with random eruption dates selected from a uniform distribution of dates from 30 to 80 ka BP were conducted per hemisphere, and the probability that the root-mean-squared best match statistic for the distribution of the actual ages was significantly less than for the randomly generated age distributions was determined. The RMS statistic provides a summation of the distances between individual eruption ages and the nearest abrupt climate change event. If no correlation exists between eruptions and climate shifts, the sum of all the root-mean-squared best match statistics should be no different than that produced using the randomly-generated eruption dates. Two sets of simulations were run, one to test the correlation between NH eruptions and abrupt Greenland cooling, and a second to test the correlation between SH eruptions and DO events (20 million simulations in total). The Monte Carlo simulations reveal that for NH eruptions, the root-mean-squared best match statistic is significantly less than the value derived from randomly-generated eruption ages, and that a 95.57% chance exists that that abrupt cooling was linked to NH volcanic eruptions (i.e., that the null hypothesis can be rejected). On the other hand, the Monte Carlo simulations reveal that SH eruptions dates are not statistically associated with DO events. This either reflects: *i)* the lack of a link between the two, or *ii)* the small number (n = 5) of large radiometrically-dated volcanic eruptions over the time interval of interest and the large uncertainties associated with their dating. Importantly, Bay et al. (2004)[31] used optical dust logger data measured in Antarctic Siple Dome ice to link SH eruptions with DO events over the period 27 to 70 ka. The link between SH volcanism and DO events was significant at the 99%

confidence level, suggesting that SH volcanism was in fact associated with DO events. However, the volcanic eruptions responsible for the Siple Dome ice signal are largely unknown, and are therefore not included in our analysis based on radiometric dates of known volcanic eruptions. We therefore suggest that the low statistical significance of the correlation between SH eruptions and DO events in our analysis is due to the very large number of unknown SH eruptions. Considered together, the results presented in our research (suggesting a ~96% likelihood of a link between NH volcanism and abrupt Greenland cooling) and those of Bay et al. (2004) (suggesting a 99% likelihood of a link between SH volcanism and abrupt Greenland warming) strongly support the conclusion that abrupt climate change during the Last Glacial was triggered by hemispheric temperature asymmetry induced by large volcanic eruptions. The climatic effects of the eruptions were then prolonged by an ice/albedo/ocean circulation positive feedback, similar to that already suggested as having sustained cool NH temperatures for hundreds of years following large 13th Century NH volcanic eruptions (and leading to the Little Ice Age)[32, 33]. The presence of large NH continental ice sheets during the Last Glacial could have amplified the effects of eruptions even more than those that occurred during the last millennium. Future research should focus on obtained high-precision dates of large Quaternary eruptions, particularly those that occurred in the SH, in order to test the hypothesis further.

References

1 Veres, D. *et al.* The Antarctic ice core chronology (AICC2012): an optimized multi-parameter and multi-site dating approach for the last 120 thousand years. *Clim. Past* **9**, 1733-1748 (2013).

2 Rasmussen, S. O. *et al.* A stratigraphic framework for abrupt climatic changes during the Last Glacial period based on three synchronized Greenland ice-core records: refining and extending the INTIMATE event stratigraphy. *Quaternary Sci. Rev.* **106**, 14-28 (2014).

3 Svensson, A. *et al.* Direct linking of Greenland and Antarctic ice cores at the Toba eruption (74 ka BP). *Clim. Past* **9**, 749-766 (2013).

4 Mark, D. F. *et al.* A high-precision Ar-40/Ar-39 age for the Young Toba Tuff and dating of ultra-distal tephra: Forcing of Quaternary climate and implications for hominin occupation of India. *Quat. Geochronol.* **21**, 90-103 (2013).

5 Storey, M. *et al.* Astronomically calibrated 40Ar/39Ar age for the Toba supereruption and global synchronization of late Quaternary record. *Proc. Natl. Acad. Sci. USA* **109**, 18684 - 18688 (2012).

6 Carolin, S. A. *et al.* Varied response of western Pacific hydrology to climate forcings over the Last Glacial period. *Science* **340**, 1564-1566 (2013).

7 Moseley, G. E. *et al.* Multi-speleothem record reveals tightly coupled climate between central Europe and Greenland during Marine Isotope Stage 3. *Geol.* **42**, 1043-1046 (2014).

8 Man, W. M. *et al.* Effects of large volcanic eruptions on global summer climate and East Asian monsoon changes during the Last Millennium: analysis of MPI-ESM simulations. *J. Clim.* **27**, 7394-7409 (2014).

9 Robock, A. Volcanic eruptions and climate. *Rev. Geophys.* **38**, 191 - 219 (2000).

10 Self, S. The effects and consequences of very large explosive volcanic eruptions. *Philos T R Soc A* **364**, 2073-2097 (2006).

11 Kravitz, B. & Robock, A. Climate effects of high-latitude volcanic eruptions: Role of the time of year. *J. Geophys. Res. - Atm.* **116** (2011).

12 Oman, L. *et al.* High-latitude eruptions cast shadow over the African monsoon and the flow of the Nile. *Geophys. Res. Lett.* **33** (2006).

13 Haywood, J. M. *et al.* Asymmetric forcing from stratospheric aerosols impacts Sahelian rainfall. *Nat. Clim. Change* **3**, 660-665 (2013).

14 Ridley, H. E. *et al.* Aerosol forcing of the position of the intertropical convergence zone since AD1550. *Nat. Geoscience* **8**, 195–200 (2015).

15 Hudson, R. D. Measurements of the movement of the jet streams at mid-latitudes, in the Northern and Southern Hemispheres, 1979 to 2010. *Atmos. Chem. Phys.* **12**, 7797-7808 (2012).

16 Olsen, J. *et al.* Variability of the North Atlantic Oscillation over the past 5,200 years. *Nat. Geoscience* **5**, 808-812 (2012).

17 Haug, G. H. *et al.* Southward migration of the intertropical convergence zone through the Holocene. *Science* **293**, 1304-1308 (2001).

18 Arbuszewski, J. A. *et al.* Meridional shifts of the Atlantic intertropical convergence zone since the Last Glacial Maximum. *Nat. Geoscience* **6**, 959-962 (2013).

19 Chiang, J. C. H. *et al.* South Pacific Split Jet, ITCZ shifts, and atmospheric North-South linkages during abrupt climate changes of the last glacial period. *Earth Planet. Sc. Lett.* **406**, 233-246 (2014).

20 Alley, R. B. Wally was right: Predictive ability of the North Atlantic "Conveyor belt" hypothesis for abrupt climate change. *Annu. Rev. Earth Pl. Sc.* **35**, 241-272 (2007).

21 Robock, A. Pinatubo eruption - The climatic aftermath. *Science* **295**, 1242-1244 (2002).

22 Bourassa, A. E. *et al.* Large Volcanic Aerosol Load in the Stratosphere Linked to Asian Monsoon Transport. *Science* **337**, 78-81 (2012).

23 Oppenheimer, C. Ice core and palaeoclimatic evidence for the timing and nature of the great mid-13th century volcanic eruption. *Int. J. Climatol.* **23**, 417-426 (2003).

24 Sun, C. *et al.* Ash from Changbaishan Millennium eruption recorded in Greenland ice: Implications for determining the eruption's timing and impact. *Geophys. Res. Lett.* **41**, 694-701 (2014).

25 Sigl, M. *et al.* Insights from Antarctica on volcanic forcing during the Common Era. *Nat. Clim. Change* **4**, 693-697 (2014).

26 Huybers, P. Early Pleistocene glacial cycles and the integrated summer insolation forcing. *Science* **313**, 508-511 (2006).

27 Brown, S. *et al.* Characterisation of the Quaternary eruption record: analysis of the Large Magnitude Explosive Volcanic Eruptions (LaMEVE) database. *J. Appl. Volc.* **3**, 5 (2014).

28 Kelley, S. K-Ar and Ar-Ar dating. *Rev Mineral Geochem* **47**, 785-818 (2002).

29 Wilson, C. J. N. *et al.* A multiple-approach radiometric age estimate for the Rotoiti and Earthquake Flat eruptions, New Zealand, with implications for the MIS 4/3 boundary. *Quat. Sci. Rev.* **26**, 1861-1870 (2007).

30 Watt, S. *et al.* The volcanic response to deglaciation: Evidence from glaciated arcs and a reassessment of global eruption records. *Earth Sci. Rev.* **122**, 77-102 (2013).

31 Bay, R. C. *et al.* Bipolar correlation of volcanism with millennial climate change. *P. Natl. Acad. Sci. USA* **101**, 6341-6345 (2004).

32 Miller, G. H. *et al.* Abrupt onset of the Little Ice Age triggered by volcanism and sustained by sea-ice/ocean feedbacks. *Geophys. Res. Lett.* **39**, L02708 (2012).

33 Schneider, D. P. *et al.* Climate response to large, high-latitude and low-latitude volcanic eruptions in the Community Climate System Model. *J. Geophys. Res-Atmos.* **114**, D15101 (2009).

34 http://riodb02.ibase.aist.go.jp/strata/VOL_JP/EN/index.htm. *Catalog of Quaternary Volcanoes in Japan*, (1999).

35 http://www.gvb-csic.es/CCDB/newsFRAM.htm. *Collapse Caldera Database*, (2011).

36 Braitseva, O. A. *et al.* Ages of calderas, large explosive craters and active volcanoes in the Kuril-Kamchatka region, Russia. *B. Volcanol.* **57**, 383-402 (1995).

37 De Vivo, B. *et al.* New constraints on the pyroclastic eruptive history of the Campanian volcanic Plain (Italy). *Miner. Petrol.* **73**, 47-65 (2001).

38 Fairbanks,R.G., et al. (2005) Marine radiocarbon calibration curve spanning 0 to 50,000 years B.P. based on paired ^{230}Th/^{234}U/^{238}U and ^{14}C dates on pristine corals, *Quat. Sci. Rev.* **24**, 1781-1796.

39 Sutawidjaja, I. S. Ignimbrite analyses of Batur caldera, Bali, based on ^{14}C Dating. *Jurnal Geologi Indonesia* **4**, 189-202 (2009).

40 Dixon, H. J. *et al.* The geology of Nevados de Chillán volcano, Chile. *Revista Geológica de Chile* **26**, 227-253 (1999).

41 Danišík, M. *et al.* Re-anchoring the late Pleistocene tephrochronology of New Zealand based on concordant radiocarbon ages and combined ^{238}U/^{230}Th disequilibrium and (U-Th)/He zircon ages. *Earth. Planet. Sc. Lett.* **349**, 240-250 (2012).

Supplementary Table Captions

Table S1. Dates of DO events discussed. Dates are consistent with other published dates, but were obtained using NGRIP $\delta^{18}O$ values on the AICC2012 chronology. The date represents the first indication of warming associated with individual DO events.

Table S2. Dates of Glacial Stadial and Interglacial abrupt cold substage initiations over the interval 30-80 ka BP. The dates are based on the observed timing of rapid cooling in the NGRIP $\delta^{18}O$ values on the AICC2012 chronology, and therefore represents the first indication of cooling associated with individual events. Event identification and nomenclature is based on the INTIMATE event stratigraphy presented in Rasmussen et al. (2014).

Table S3. Radiometrically dated volcanic eruptions used in this research. All M ≥ 6 SH eruptions with radiometric ages and all M ≥ 7 NH eruptions featured in the LaMEVE database are listed, along with the reference for the radiometric date[3, 35-41]. In the case of Uzon, Okataina I (Mangaone), and Okataina K, we have recalculated the radiocarbon dates using a more recent calibration curve (Fairbanks et al., 2005), and the dates in the table are therefore different than the ones presented in LaMEVE.

Supplementary Tables:

DO event	Initiation age(ka BP)
DO-4	28.85
DO-5.1	30.79
DO-5.2	32.45
DO-6	33.69
DO-7	35.43
DO-8	38.17
DO-9	40.11
DO-10	41.41
DO-11	43.29
DO-12	46.81
DO-13	49.23
DO-14	54.17
DO-15.1	54.95
DO-15.2	55.75
DO-16.1	57.99
DO-16.2	58.23
DO-17.1	59.03
DO-17.2	59.39
DO-18	64.05
DO-19.1	69.57
DO-19.2	72.29
DO-20	75.89

Table S1

**Greenland abrupt cooling event**	**Initiation age (ka BP)**
GS-5.1	30.55
GS-6	33.31
GS-7	34.69
GI-7b	34.97
GS-8	36.53
GI-8b	37.07
GS-9	39.85
GS-10	40.75
GS-11	42.19
GS-12	44.23
GI-12b	44.63
GS-13	48.29
GI-13b	49.07
GS-14	49.55
GI-14d	53.97
GS-15.1	54.85
GS-15.2	55.35
GS-16.1	56.45
GI-16.1b	57.91
GS-16.2	58.11
GS-17.1	58.51
GI-17.1b	58.79
GS-17.2	59.25
GS-18	63.79
GS-19.1	69.35
GS-20	73.7729
GI-20b	74.39

Table S2

VOLCANO NAME	RADIOMETRIC AGE OF ERUPTION (ka BP)	DATING ERROR (ka BP)	reference(s)	technique
**Northern Hemisphere**				
Shikotsu	37.376	1.988	Catalog of Quaternary Volcanoes in Japan[34]	radiocarbon
Bulusan	37.5	2.5	Collapse Caldera Database, 2011[35]	Ar-Ar
Gorely	38.981	0.704	Braitseva et al. 1995[36]	radiocarbon
Campi Flegrei	39.28	0.11	De Vivo et al., 2001[37]	Ar-Ar
Uzon	44.375	1.798	Fairbanks et al. 2005[38]	radiocarbon
Opala	44.902	0.725	Braitseva et al. 1995[36]	radiocarbon
Ischia	53	3.3	Collapse Caldera Database, 2011[35]	Ar-Ar
Toba	73.75	0.32	Svennson et al., 2013[3]	ice core
**Southern Hemisphere**				
Okataina (Unit I; Mangaone)	33.428	0.323	Fairbanks et al. 2005[38]	radiocarbon
Okataina (Unit K)	33.592	0.48	Fairbanks et al. 2005[38]	radiocarbon
Batur	34.565	3.682	Sutawidjaja 2009[39]	radiocarbon
Nevados de Chillán	42.565	0.463	Dixon et al., 1999[40]	radiocarbon
Okataina	45.1	3.3	Danǐsǐk et al., 2012[41]	U-series / He

Table S3
